# Hidden cause of paralysis: tight filum terminale in spinal cord injury without radiographic abnormality

**DOI:** 10.3389/fped.2024.1528007

**Published:** 2025-01-22

**Authors:** Yi Yuan, Zong Jian An, Fei Gao, Zhi Hui Li, Wei Li Xu, Yong Sun

**Affiliations:** ^1^ School of Clinical Medicine, Shandong Second Medical University, Weifang, China; ^2^ Woman and Children’s Hospital, Qingdao University, Qingdao, China

**Keywords:** children, spinal cord injury, tethered cord, surgery, spinal cord injury without radiographic abnormality (SCIWORA)

## Abstract

**Objective:**

To explore the clinical characteristics and surgical treatment outcomes of spinal cord injury without radiographic abnormality (SCIWORA) in children.

**Methods:**

A retrospective analysis was conducted on the clinical data of four children diagnosed with SCIWORA who were admitted to the Neurosurgery Department of Qingdao Women and Children's Hospital from November 2022 to June 2024. All four pediatric patients underwent laminectomy for spinal canal decompression along with resection of the filum terminale. Postoperatively, a regimen including corticosteroids and mannitol was administered. Following hospital discharge, each child was subjected to clinical follow-ups, and the neurological recovery from spinal cord injury was evaluated utilizing the American Spinal Injury Association (ASIA) impairment scale.

**Results:**

Among the four patients, there was one male and three females, aged from 3 years and 2 months to 8 years. Two cases were due to low falls, one from a lower back injury, and one from a lumbar sprain. All patients had thoracolumbar injuries, with rapid progression of symptoms, including paralysis, sensory impairment, and urinary and fecal retention. Follow-up duration ranged from 3 to 24 months; three patients showed varying degrees of recovery in muscle strength and/or sensory function and bowel and bladder control, while one showed no improvement. One patient developed scoliosis and another presented with neurogenic bladder.

**Conclusion:**

Tethered cord syndrome may be a potential underlying cause of SCIWORA. For children with SCIWORA accompanied by tethered cord syndrome, we recommend early surgical intervention to perform laminectomy and release the tethered cord, which may aid in the recovery of neurological function.

## 1 Introduction

Spinal cord injury without radiographic abnormalities (SCIWORA) was first proposed by Burke in 1974 (1) and subsequently defined by Pang and Wilberger in 1982 as an acute spinal cord injury (TSCI) (2) caused by trauma, with no evidence of vertebral fractures or dislocations on x-ray or CT scans. SCIWORA accounts for 13%–42% of all pediatric spinal cord injuries, with the majority of cases occurring in children aged 3–10 years (3). This study retrospectively analyzed the clinical data of four SCIWORA patients treated at our hospital from November 2022 to June 2024, exploring their injury mechanisms, clinical features, spinal cord imaging changes, treatment, and prognosis to provide a reference for the management of SCIWORA.

## 2 Subjects and methods

Clinical data of children diagnosed with SCIWORA admitted to our hospital from November 2022 to June 2024 were collected, including age, gender, cause of injury, injury site, diagnostic imaging methods, treatment strategies, neurological function grading, and prognosis.

Upon emergency admission, all patients underwent neurological examinations to assess their motor and sensory functions. The neurological status was evaluated according to the American Spinal Injury Association (ASIA). classification. x-ray and CT were used to assess fractures, subluxations, dislocations, deformities, and soft tissue injuries. Routine magnetic resonance imaging (MRI) was further used to evaluate spinal cord and soft tissue injuries.

## 3 General information

**Figure 1:**
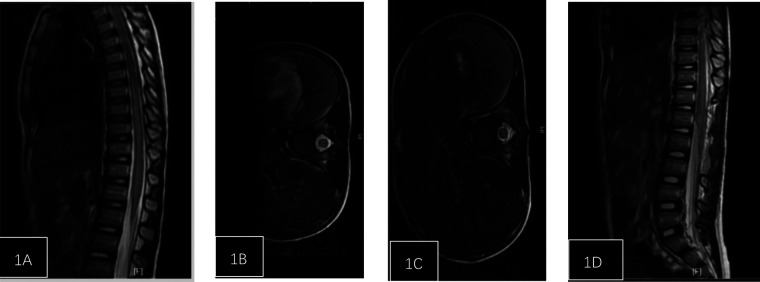
Patient, female, 5 years and 1 month, injured from a fall. **(A)** Post-injury spinal MRI shows edema of the spinal cord from T6 to L2 and dilatation of the central canal. **(B)** Axial T2-weighted imaging shows high signal intensity. **(C)** One week post-surgery follow-up spinal MRI shows reduced edema compared to pre-treatment, with no central canal dilation observed. **(D)** Axial T2-weighted imaging.

**Figure 2:**
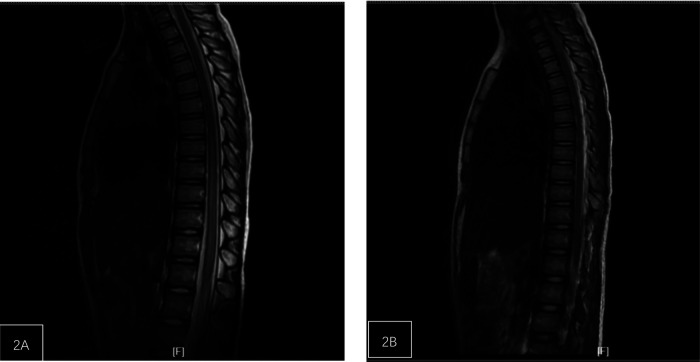
Patient, female, 8 years old, with lower back injury. **(A)** The spinal cord at the T7-L1 level is slightly enlarged with abnormal signal. **(B)** One-week post-operation, the range of abnormal signals in the spinal cord at the t7-L1 level has significantly decreased.

**Figure 3:**
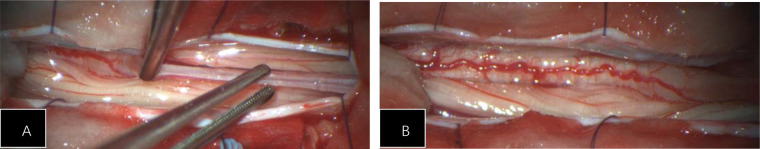
**(A)** During the surgery, significant edema of the conus medullaris was observed, with thickening and high tension of the filum terminale. **(B)** The spinal cord is congested and edematous, with poor pulsation.

All four cases had a clear history of thoracolumbar trauma, with three females and one male; their ages were 3 years and 2 months, 5 years and 1 month, 5 years and 6 months, and 8 years (Table 1). The mechanisms of injury were: one case of lower back injury, one case of lumbar sprain, and two cases of low falls. Before the injuries, none of the patients exhibited any neurological dysfunction. Two patients experienced immediate symptoms of lower limb weakness, sensory loss, and bladder and bowel dysfunction, while the other two developed symptoms a few hours to a few days post-injury. All four patients underwent decompression surgery and termination of the filum terminale. During surgery, significant edema of the conus medullaris and thickening of the filum terminale were observed (Figures 1, 2, 3).

**Table 1:** General information.

Project	No. 1	No. 2	No. 3	No.4
Gender	Female	Female	Female	Male
Age	8 years	5 years 6 months	5 years 1 month	3 years 2 months
Injury mechanism	Lower back	low fall	low fall	Lumbar sprain
Main clinical manifestations	Bilateral lower limb muscle strength grade I, hypersensitivity in lower limbs, urinary retention, weak anal reflex	Weakness in lower limbs, muscle strength grade 0, sensory loss, urinary retention	Motor impairment in lower limbs, muscle strength grade I, urinary retention	Weakness in lower limbs with limited movement, numbness in lower limbs, muscle strength grade III in left lower limb, grade II in right lower limb, difficulty urinating
Time symptoms appeared	10 min	Immediately	2 days post-injury	immediately
Consultation Time	16 h Post-injury	6 h Post-injury	2 days Post-injury	7 h Post-injury
Initial spinal cord injury ASIA Classification	A	A	B	C
MRI Findings	Spinal cord slightly enlarged with abnormal signals at T7-L1, sacral cleft below S1	Central canal enlargement of spinal cord at T6-L2	Abnormal signals in the spinal cord below T8, slightly high signal in lumbar spinal cord	Low position of the conus medullaris, lumbarization of S1 vertebra
Surgery time	26 h post-injury	23 h post-injury	54 h post-injury	9 h post-injury
Treatment method	Decompressive laminectomy + filum terminale section	Decompressive laminectomy + filum terminale section	Decompressive laminectomy + filum terminale section	Decompressive laminectomy + filum terminale section
Follow-up ASIA classification	D	A	D	E
Follow-up prognosis	15 months follow-up, abnormal walking posture, secondary scoliosis, occasional urinary urgency, normal bowel movements	3 months follow-up, muscle strength grade I in lower limbs, long-term indwelling catheter, regular bowel movements with suppositories	11 months follow-up, neurogenic bladder, normal bowel movements	6 months follow-up, normal muscle strength and tone in lower limbs, normal urination and defecation

## 4 Follow-up results

Over a follow-up period of 3–24 months, three patients showed varying degrees of recovery in muscle strength and/or sensory function, as well as bladder and bowel function, while one showed no significant improvement. One patient developed scoliosis, and another developed neurogenic bladder.

## 5 Discussion

The incidence of SCIWORA in children is between 8% and 32%, with rates of 9.4%in the 3–12 age group and 5% in the 13–20 age group (4). In a meta-analysis by Jazayeri et al., traffic accidents were the most common cause of TSCI injuries in children from developed countries, primarily involving cervical spinal cord injuries, while in developing countries, sports-related injuries were most common, primarily affecting the thoracic spinal cord (5), In this group, all four cases involved thoracolumbar injuries, with ages ranging from 3 to 8 years, and the injuries were caused by accidental trauma to the lumbar region. Intraoperative findings in all four pediatric cases revealed spinal cord tethering, with evidence of fatty or fibrous degeneration involving the filum terminale.

SCIWORA typically occurs following excessive extension of the spine. Early symptoms may include pain, numbness, or weakness in the lower back or legs. Although most children can stand and walk immediately after the injury, symptoms may rapidly worsen within hours, presenting with typical spinal cord injury symptoms below the level of injury, such as loss of sensory and motor function, as well as urinary and bowel dysfunction. The level of neurological damage is often between T4 and T12, with the most common lesions located above and below T10 (6). In this group, symptoms resulted in paralysis below the T10 level, accompanied by incontinence.

The mechanisms of SCIWORA injuries include longitudinal traction, excessive flexion, excessive extension, direct compression injuries, or a combination of these, as well as ischemic spinal cord injuries (7).

Spinal Cord Tethering Syndrome (TCS) encompasses a spectrum of skeletal, neurological, and urogenital symptoms due to the fixation of inelastic tissue on the conus medullaris, resulting in cord stretching and restricted mobility within the spinal canal. Characteristic findings on lumbar magnetic resonance imaging (MRI) may include a low-lying conus medullaris or a pathologic filum terminale that is thickened and/or fatty. MRI often reveals additional pathologies, such as congenital or acquired lumbosacral anomalies. The emergence of TCS symptoms typically coincides with pediatric growth spurts, attributed to the disparity in growth rates between the musculoskeletal components of the spinal column and the neuroaxis, leading to excessive strain on the tethered spinal cord (8).

The elasticity of the pediatric spine is greater than that of the spinal cord; under the same external force, the spine may not show obvious damage, while the spinal cord could suffer severe injury (9). Excessive spinal extension, such as during backward bending, can result in vascular damage, bleeding, and edema in the spinal cord, potentially causing obstruction of venous return. This may lead to increased venous pressure in the spinal cord and a decrease in the arterial-venous pressure gradient (10). This mechanism could be due to compression of the inferior vena cava by abdominal organs, coupled with increased pressure in the epidural venous plexus caused by the Valsalva maneuver, collectively exacerbating the obstruction to venous outflow from the spinal cord. Such venous return obstruction may further trigger insufficient spinal cord blood perfusion, venous thrombosis, and increased vascular permeability, damaging the blood-spinal cord barrier, thereby exacerbating secondary spinal cord injury and, in severe cases, leading to spinal cord infarction and atrophy (11,12). Furthermore, spinal cord injury (SCI) can interfere with the normal cerebrospinal fluid (CSF) circulation or impact the microcirculation within the spinal cord parenchyma, leading to ischemia and localized hypoxia. Chen et al. reported that among 74 pediatric SCI cases, 20 had occult spinal dysraphism, with 4 exhibiting fatty degeneration of the filum terminale (13). Liang et al. (7) reported three pediatric cases of SCIWORA complicated by fibrofatty filum terminale, suggesting that tight filum terminale (TFT) may be a predisposing factor for SCIWORA. They emphasized that chronic traction on the spinal cord could play a significant role in the pathogenesis of thoracolumbar SCIWORA in children after minor trauma. Patients who have not received treatment for TFT may be at higher risk for developing SCIWORA following minor trauma. Yuan et al. (14) also reported on eight cases of SCIWORA, suggesting that the presence of filum terminale syndrome, a form of tethered cord, may be associated with SCIWORA, and that early sectioning of the filum terminale might potentially reduce the risk of further spinal cord injury.

Additionally, even with the conus medullaris in a normal position, tethered cord syndrome cannot be ruled out, as tension in the filum terminale may lead to longitudinal traction on the spinal cord. This traction could exert pressure and cause injury to the spinal cord during body movements, especially during spinal flexion (15). Literature indicates that in asymptomatic SCI patients, over 50% of radiological findings may show suspected tethered cord syndrome and/or early signs of syringomyelia (16). Abnormalities in the filum terminale, such as increased width, lipomas, or fibrosis, may lead to a loss of elasticity, resulting in anchoring of the conus medullaris and pulling on the caudal portion of the spinal cord, worsening spinal cord injury (17). In this group, all four pediatric cases had fatty degeneration of the filum terminale confirmed during surgery. Preoperative MRIs did not show thickening of the filum terminale, but the conus medullaris displayed significant edema and morphological abnormalities, indicating some traction affecting the spinal cord. Therefore, we believe that even when the conus medullaris appears in a normal plane on imaging, it is essential to carefully evaluate the morphology of the conus, the filum terminale, and other spinal structures to determine if any biomechanical issues may lead to spinal cord injury, warranting timely surgical intervention.

The treatment of SCI in children and adolescents remains controversial. Current standard treatments include high-dose steroid pulse therapy and surgery. There is ongoing controversy regarding the safety of steroid treatment, and some literature indicates that high-dose methylprednisolone is not associated with improved outcomes (18). The biological principle behind early surgical decompression after acute spinal cord injury is to mitigate secondary damage. Data suggest that surgical decompression post-injury may alleviate secondary damage and improve neurological outcomes, with effects inversely related to the duration of compression (19, 20). Some scholars advocate for decompression surgery only in cases of spinal cord compression with neurological deficits, but a meta-analysis by Batchelor showed that early decompression improved functional outcomes by 35.1% (21). In a rat model of SCIWORA by Okimatsu et al., both acute and subacute decompression surgeries were effective, with earlier surgeries leading to faster recovery (22).

It has been reported that patients receiving early decompression within 24 h of SCI have 2.8 times greater likelihood of achieving at least a 2-level improvement on the ASIA impairment scale (AIS) at six months compared to those undergoing late decompression (≥24 h post-SCI). Patients with early decompression classified as AIS B, C, and D showed an additional 6.3 points increase in motor recovery compared to late decompression patients (23). Badhiwala et al. conducted a pooled analysis of individual patient data from four multicenter studies on spinal cord injury conducted between 1991 and 2017 (*n* = 1,548). They found that patients undergoing early decompression (within 24 h of injury) showed significantly faster recovery of motor and sensory functions compared to those receiving late surgery (≥24 h post-injury) and noted that surgery within 36 h post-SCI may still be beneficial (24).

In this case series, four pediatric patients received proactive surgical intervention within 48 h of symptom onset, and all cases confirmed the presence of spinal cord tethering intraoperatively. Postoperative MRI at one week showed a notable reduction in the extent of abnormal spinal cord signals. We believe that timely surgery, complete decompression, and resolution of tethering, followed by duroplasty to expand the spinal canal, can significantly alleviate intraspinal pressure and enhance spinal cord perfusion. Duroplasty improves radiological and physiological parameters by expanding the space around the injured spinal cord, reducing intra-spinal pressure (ISP), and increasing spinal cord perfusion pressure (SCPP), thus mitigating or alleviating secondary spinal cord injuries. This strategy can reduce the incidence of neurologic deficits, improve outcomes, and enhance prognosis. Except for one child with no significant improvement in this series, the remaining patients had favorable outcomes. We consider the lack of improvement in this case may be attributed to a short follow-up period and preoperative MRI indicating syringomyelia, suggesting more severe spinal cord injury and prolonged traction ischemia.

Spinal deformities are common complications, especially in children with skeletal dysplasia. Prior to peak growth, 97% of children with SCI develop scoliosis, and being younger than 14.6 years is a predictive factor for scoliosis (25). Urinary dysfunction is a major cause of disability and mortality in young patients, who may also experience hip abnormalities, osteoporosis, and hypercalcemia. In our group, complications included scoliosis and neurogenic bladder, necessitating further rehabilitation (26).

This study acknowledges several limitations. Firstly, due to the rarity of SCIWORA, the sample size evaluated in this study was relatively small and the follow-up period was short-term. Secondly, while the study presents outcomes of surgical decompression, it lacks a comparative analysis with conservative treatment approaches. Therefore, the findings presented here will necessitate validation through a comparative, prospective study.

In summary, tethered cord syndrome may be a potential underlying cause of SCIWORA. For children with SCIWORA accompanied by tethered cord syndrome, we recommend early surgical intervention for decompression and simultaneous release of the tether, which can aid in the recovery of neurological function.

## Data Availability

The original contributions presented in the study are included in the article/Supplementary Material, further inquiries can be directed to the corresponding author.
